# Integrated analysis of the roles of oxidative stress related genes and prognostic value in clear cell renal cell carcinoma

**DOI:** 10.1007/s00432-023-04983-w

**Published:** 2023-06-20

**Authors:** Danwen Wang, Zhao Deng, Mengxin Lu, Kai Deng, Zhiqiang Li, Fenfang Zhou

**Affiliations:** 1 Department of Neurosurgery, Zhongnan Hospital, Wuhan University, Wuhan, China; 2 Department of Urology, Zhongnan Hospital of Wuhan University, Wuhan, China; 3 Department of Radiology, Zhongnan Hospital of Wuhan University, Wuhan, China

**Keywords:** ccRCC, OSRGs, ROS, PYCR1, MELK

## Abstract

**Background:**

Patients with clear cell renal cell carcinoma (ccRCC), which is the most commonly diagnosed subtype of renal cell carcinoma, are at risk of tumor metastasis and recrudescence. Previous research has shown that oxidative stress can induce tumorigenesis in many cancers and can be a target of cancer treatment. Despite these findings, little progress has been made understanding in the association of oxidative stress-related genes (OSRGs) with ccRCC.

**Methods:**

In vitro experiments were conducted with MTT survival assays, qRT‒PCR, apoptosis assays, cell cycle assays, ROS assays, and IHC staining.

**Results:**

In our study, 12 differentially expressed oxidative stress-related genes (DEOSGs) and related transcription factors (TFs) that are relevant to overall survival (OS) were screened, and their mutual regulatory networks were constructed with data from the TCGA database. Moreover, we constructed a risk model of these OSRGs and performed clinical prognostic analysis and validation. Next, we performed protein–protein interaction (PPI) network analysis and Gene Ontology (GO) and Kyoto Encyclopedia of Genes and Genomes (KEGG) pathway enrichment analysis of MELK, PYCR1, and PML. A tissue microarray also verified the high expression of MELK and PYCR1 in ccRCC. Finally, in vitro cellular experiments demonstrated that knockdown of MELK or PYCR1 significantly inhibited ccRCC cell proliferation by causing cell apoptosis and inducing cell cycle arrest in the G1 phase. Intracellular ROS levels were elevated after these two genes were knocked down.

**Conclusion:**

Our results revealed the potential DEORGs to be used in ccRCC prognostic prediction and identified two biomarkers, named PYCR1 and MELK, which regulated the proliferation of ccRCC cells by affecting ROS levels. Furthermore, PYCR1 and MELK could be promising targets for predicting the progression and prognosis of ccRCC, thereby serving as new targets for medical treatments.

**Supplementary Information:**

The online version contains supplementary material available at 10.1007/s00432-023-04983-w.

## Introduction

Renal cell carcinoma, a tumor characterized by high incidence and mortality rates, poses a gradually increasing threat to human health (Siegel et al. 2017). ccRCC is the most common histological subtype of renal cell carcinoma, and it accounts for more than 80% of renal cell carcinoma cases (Barth et al. 2019). Recently, cancer genome studies have revealed the great intra- and intertumor heterogeneity of ccRCC, and its clinical symptoms and prognosis are even more elusive to physicians (Hsieh et al. 2017). For patients with early, localized cancer, the five-year survival rate can reach 90% after treatment with current therapeutic methods, including TKIs, cabozantinib, and immunotherapy. In contrast, five-year survival decreases to 12% for patients with distant metastatic disease (Atkins and Tannir 2018). Although the use of targeted molecular therapy in ccRCC patients has shown remarkable achievements, the clinical efficacy is not yet satisfactory for patients with tumor recurrence or metastasis (Yang and Chen 2020). Therefore, it is critical to explore more effective diagnostic markers and novel strategies to improve therapeutic effects in patients with advanced ccRCC.

Oxidative stress, which is a universal pathological phenomenon, basically refers to an excess of reactive oxygen species (ROS) relative to antioxidants (Hayes et al. 2020). In normal cells, when the antioxidant defense system is insufficient for coping with the damage caused by oxidative stress, continued exposure to high levels of ROS can damage intracellular components such as DNA and thus increase the risk of carcinoma (Rose et al. 2020; Gill et al. 2016). In contrast, in cancer cells, ROS affects many aspects of tumor progression, including cell proliferation, evasion of apoptosis or anoikis, invasion and metastasis, and angiogenesis (Sosa et al. 2013). Notably, oxidative stress in cells can lead to the disruption of the intracellular redox balance (Abu et al. 2017; Day et al. 2012). This disrupted balance contributes to the excessive accumulation of ROS, which can cause clear damage to the cellular structure, disrupting cell metabolism and destroying nucleic acid stability, thereby causing cell death (Prasad et al. 2017). Recent research has concluded that the induction of oxidative stress can preferentially kill cancer cells due to the higher sensitivity of cancer cells to further accumulation of ROS (Trachootham et al. 2009). Various drugs that direct target ROS metabolism are already available in clinical medicine, such as NOV-002, sulfasalazine and celecoxib (Trachootham et al. 2009). The level of ROS is significantly elevated in renal cancer patients, and previous research has shown that cellular redox homeostasis changes significantly in ccRCC (Ganesamoni et al. 2012; Pelicano et al. 2004). Previous research demonstrated that coadministration of 5-FU and belinostat increased ROS and DNA damage, and this combination can be used in the clinical treatment of metastatic renal cell carcinoma (Kim et al. 2015). Overall, substantial research has provided insights into changes in oxidative stress that play crucial roles in the progression of renal cell carcinoma. Oxidative stress has been proven to be a significant target in the treatment of cancer. However, little progress has been made in understanding the relationship of OSRGs with ccRCC. Accordingly, identifying potential novel OSRGs is the top priority for ccRCC research.

Notably, the changing characteristics of the tumor microenvironment (TME) are closely associated with the tumorigenesis, progression, and metastasis of tumors via various mechanisms. The TME consists of tumor cells and surrounding noncancerous components (including extracellular matrix, inflammatory cells, and immune cells) (Lai et al. 2021). There is increasing evidence that the components of the TME can cause oxidative stress in infiltrating immune cells, and low immunogenicity of tumor cells can impair the effect of anticancer drugs and play a significant role in tumor immune escape (Sheng et al. 2017). To cope with the decrease in oxygen levels in the TME, tumor cells generally undergo metabolic and oxidative stresses, which can restrict tumor growth but can also lead to an increase in the degree of malignancy by altering the tumor phenotype (Luis et al. 2021; Sanna and Rofstad 1994). In solid tumors, ROS are not only produced by tumor cells under oxidative stress but also can be produced by activated immune cells, such as neutrophils and macrophages (Meng et al. 2021). Increasing evidence suggests that elevated ROS levels in the TME could limit the killing ability of effector immune cells, which is strongly associated with the generation of an immunosuppressive tumor environment (Scortegagna et al. 2003). Therefore, in-depth exploration of the correlation between immune cell infiltration in the TME and oxidative stresses in ccRCC will be helpful for further identifying reliable molecular markers as well as novel strategies that target the tumor redox balance.

In this study, we screened 12 DEORGs and constructed a predictive risk model derived from the TCGA database. Then, we chose two key genes, PYCR1 and MELK, that may be potential oxidative stress-related biomarkers. Finally, we verified the transcription and protein expression levels of these two genes in clinical tumor tissues and conducted in vitro experiments to determine the role of PYCR1 and MELK in immune infiltration and OS in ccRCC, thereby determining the mechanism by which these genes affect tumor progression.

## Methods and materials

### Raw data and filtering

The transcription profiling data of 611 ccRCC samples (72 normal samples and 539 tumor samples) and clinical data were obtained from the TCGA database. The clinical data that contained invalid information were deleted. Ninety-seven OSRGs were acquired from the Gene Set Enrichment Analysis (GSEA) database. Transcription factors (TFs) were obtained from the Cistrome Cancer database (http://www.cistrome.org/).

### Differential gene expression analysis

The “limma” package was used to analyze the expression of OSRGs. |log2-fold change (FC)| equal to or greater than 1 and an adjusted P value less than 0.05 were considered the thresholds for identifying statistically significant differentially expressed used (DEGs). The R packages “pheatmap” and “ggplot2” were applied to visualize the results.

### Estimation of risk model

The “survival” package in R was used to perform univariate Cox regression analysis and multivariate Cox regression analysis. The survival analysis was performed via the R packages “survival” and “survminer”. Then, the survival curve was generated with the Kaplan–Meier method. The “survivalROC” and “timeROC” packages in R were used to graph the ROC curve. The nomogram model was plotted via the “rms” package in R.

### Enrichment analysis

The STRING database (http://string-db.org/) was used for predicting a potential protein binding network. The GO and KEGG pathway enrichment analysis were performed through the R package “ggplot2” and“clusterProfiler”.

### Cell culture and siRNA transfection

The ACHN cell line was cultured in MEM, and the Caki-1 cell line was cultured in McCoy’s 5A medium. Necessarily, all media were supplemented with 10% fetal bovine serum (FBS). The cell lines were obtained from the Cell Bank of the Chinese Academy of Science and were authenticated and tested for mycoplasma contamination before use. The siRNAs were obtained from GenePharma. The sequences of the siRNAs (*PYCR1* and *MELK*) are as follows:*siPYCR1*: 5′-GCCACAGUUUCUGCUCUCATT-3′; antisense 5′-UGAGAGCAGAAACUGUGGCTT-3′. *siMELK*: 5′-CCUGGAUCAUGCAAGAUUATT-3′;antisense 5′-UAAUCUUGCAUGAUCCAGGTT-3′; siNC 5′-UUCUCCGAACGUGUCACGUTT-3′. Lipofectamine 3000 (Invitrogen, L3000015) was used to transfect cells when cell confluence reached 30%.

### RNA extraction and qRT‒PCR

The RNA extraction assay was performed using an RNeasy mini kit (Qiagen) at 4 °C. Next, the RNA (1 μg) was reverse transcribed to cDNA. qRT‒PCR was then performed with SYBR Green PCR Master Mix according to the manufacturer's instructions. The relevant primer data sequences were as follows:

PYCR1: 5′-TGGCTGCCCACAAGATAATGG-3′; 5′-CGTGACGGCATCAATCAGGT-3′.

MELK: 5′-TCTCCCAGTAGCATTCTGCTT-3′;

5′-TGATCCAGGGATG GTTCAATAGA-3′.

GAPDH: 5′-GGAGCGAGATCCCTCCAAAAT-3′;

5′-GGCTGTTGTCATACTTCTCATGG-3′.

### MTT assay

For the MTT assay, ACHN and Caki-1 cells were seeded in a 96-well plate at 1 × 10^3^ cells per well and cultured overnight. After culturing for the indicated time, 3-(4,5-dimethylthiazol-2-yl)-2,5-diphenyltetrazolium bromide (MTT, 5 mg/ml) was added to each well and incubated for 4 h at 37 ℃. Then, the supernatants were discarded, and 200 µl of DMSO was added to each well. After shaking for 30 min, the absorbance at 540 nm was measured with a microplate reader (Bio-Rad, USA) to measure cell viability.

### Analysis of ROS production, cell cycle, and apoptosis by flow cytometry

Cellular ROS levels were measured using flow cytometry (Beckman, USA). Briefly, ACHN and Caki-1 cells were incubated with 10 µM 2′,7′-dichlorodihydrofluorescein diacetate (Sigma‒Aldrich, USA) for 30 min at 37 °C. Next, the cells were washed three times with PBS and analyzed by flow cytometry. To analyze cell apoptosis, cells were stained with Annexin V FITC Apoptosis Assay Kit I (Sungene Biotech, China) according to the instructions, followed by flow cytometry. For the cell cycle assay, cells were stained with 1000 µl of 1 × DNA Staining Solution and 10 µl of permeabilization solution (Multi sciences, China).

### Immunohistochemical (IHC) staining and tissue microarray

First, paraffin sections were incubated in citrate buffer to retrieve the antigens. Then, the sections were incubated in 3% H_2_O_2_. Then, they were incubated with the appropriate anti-MELK (Abclonal, A3530) or anti-PYCR1 (Abclonal, A13346) primary antibody followed by a secondary antibody (Anti-Rabbit-IgG H&L-HRP; Goat; Abcam, USA, cat. no. ab205718). Finally, DAB chromogen solution and HRP substrate solution were used to block the sections.

The ccRCC tissue and matched margin tissue combination tissue microarrays were purchased from Wuhan Baiqiandu Technology Co., Ltd. Specifically, the details of each sample tissue microarray are described in Supplementary Table 1. The MELK and PYCR1 protein levels were determined by calculating the percent of staining [i.e., 0 (0–5%), 1 (6–25%), 2 (26–50%), 3 (51–75%), and 4 (> 75%)] and the intensity of staining [i.e., 0 (negative), 1 (weak), 2 (moderate) and 3 (strong)] in each sample. A final immunoreactivity score (IRS) was obtained by adding the percentage and intensity scores.

### Statistical analysis

All the results were obtained from more than three independent experiments. We performed survival analysis by means of the Kaplan–Meier estimate method and counted via the log-rank test. We used GraphPad Prism 7 (USA) statistical software and R software to analyze differences between groups by two-tailed t test. Statistical significance was displayed as follows: ns = not statistically significant, *p < 0.05, **p < 0.01, ***p < 0.001.

## Results

### Identification of DEOSGs and construction of prognostic model

**Fig. 1:**
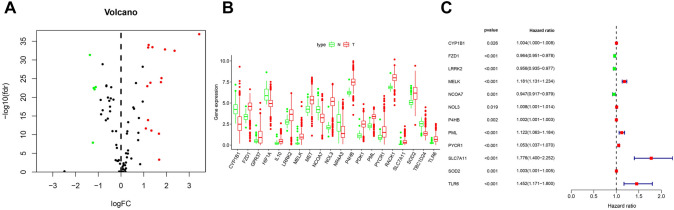
Identification of DEOSGs and prognostic model construction. **A** Volcano plot of OSRGs between TCGA-ccRCC and normal renal samples. **B** The mRNA expression levels of 20 DEOSGs in TCGA cohort. **C** Univariate Cox regression analysis for identifcation prognosis-associated OSRGs

Volcano plots were generated to visualize the expression of DEOSGs in tumor tissues compared with normal tissues. The upregulated genes are indicated by red dots, while downregulated genes are represented by green dots (Fig. 1A). According to the DEG expression analysis, FZD1, GPR37, IL10, LRRK2, MELK, MET, NOL3, P4HB, PDK1, PML, PYCR1, RACK1, SOD2, and TLR6 were highly expressed in ccRCC neoplastic tissues compared with peritumoral tissues (*P* < 0.05, Fig. 1B). Moreover, univariate Cox regression analysis showed that CYP1B1, FZD1, LRRK2, MELK, NCOA7, NOL3, P4HB, PML, PYCR1, SLC7A11, SOD2, and TLR6 were related to prognosis and could be potential risk factors for ccRCC patients (*P* < 0.05, Fig. 1C).

### Construction of the risk model of DEOSGs

**Fig. 2:**
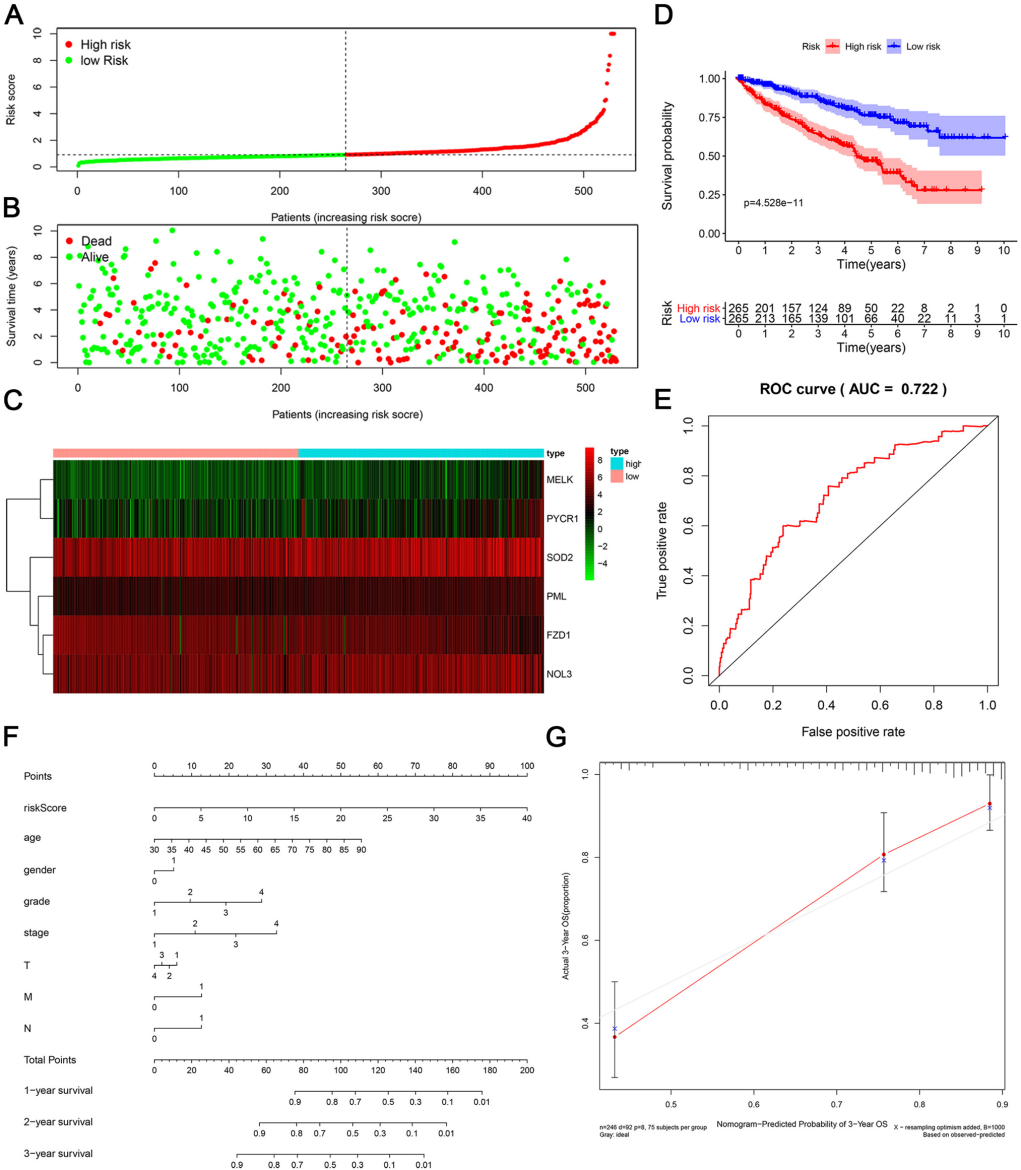
Construction of risk model of DEOSGs. **A**, **B** Risk score distribution and survival status of TCGA ccRCC patient cohort. **C** Differential expression level of 6 DEOSGs between high- and low-risk groups in the heat map. **D** Kaplan-Meier survival of TCGA cohort. **E** The ROC curves for OS in TCGA cohort. **F** Nomogram of risk score and many clinical factors for predicting ccRCC 1-, 2-, and 3-year OS in TCGA cohort. **G** The calibration charts of the 3-year nomogram in TCGA cohort

We developed a prognostic risk model according to patient survival status to further identify potential biomarkers for ccRCC patients. Patients were classified into high-risk and low-risk groups based on the risk score (Fig. 2A). Patients in the high-risk group tended to have a shorter lifespan (Fig. 2B). The heatmap shows the differential expression levels of 6 DEOSGs between the high- and low-risk groups (Fig. 2C). The Kaplan‒Meier survival curve suggested that patients with a lower risk score had a better survival rate than high-risk patients (Fig. 2D). Then, we generated an ROC curve chart to confirm the prognostic accuracy of the constructed risk model. The area under the curve (AUC) of the risk model was 0.722, indicating that it has superior predictive accuracy (Fig. 2E). Finally, to more accurately predict patient outcomes, we also constructed a prognostic nomogram from the TCGA dataset to determine the 1-, 2-, and 3-year OS for ccRCC patients, which included diverse prognostic parameters, such as risk score, age, sex, and TNM staging (Fig. 2F). The calibration charts showed that the 3-year OS predictions were highly related to the actual observations (Fig. 2G).

### Gene regulatory network of TFs and functional enrichment analysis of DEOSGs

**Fig. 3:**
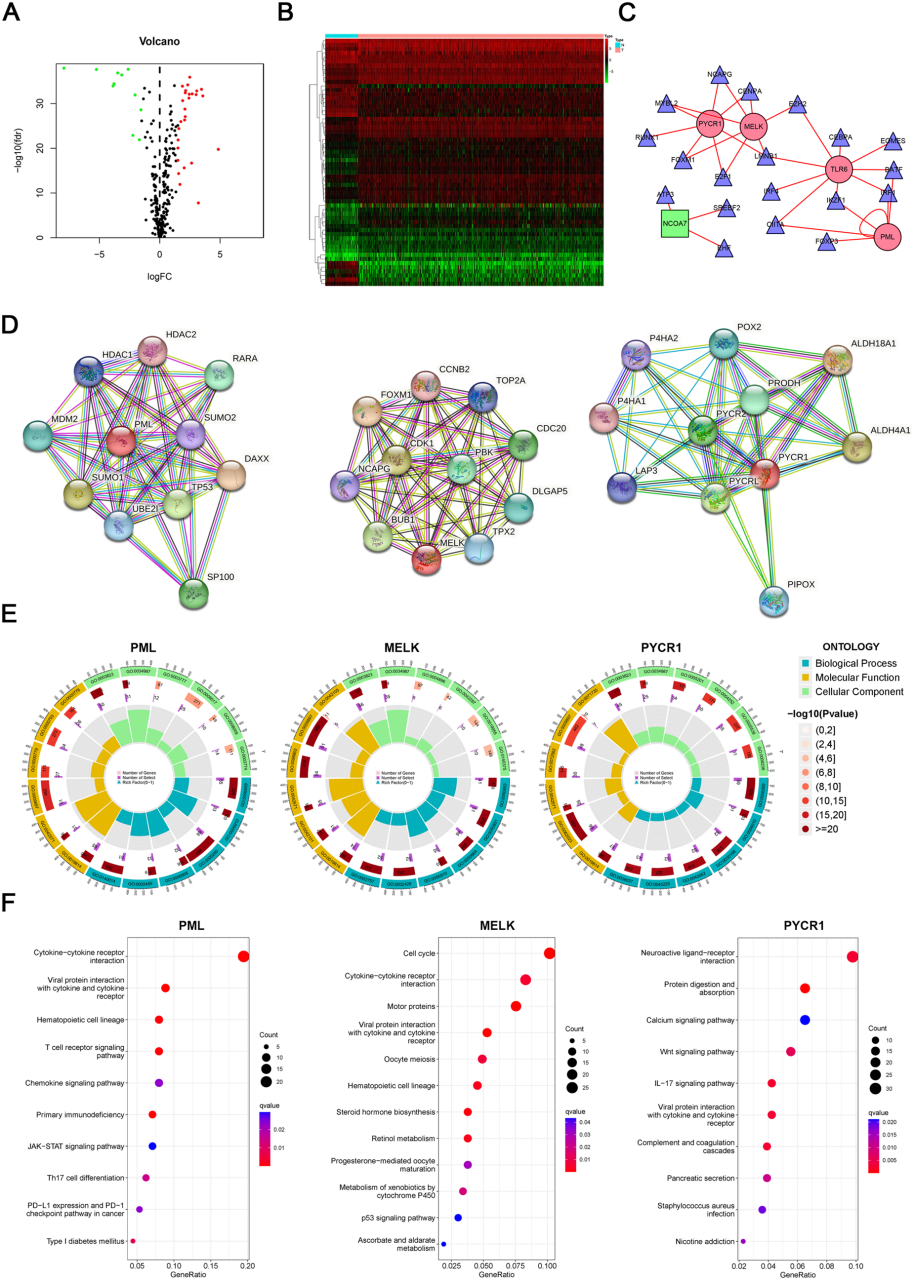
Gene regulatory network of TFs and functional enrichment analysis of DEOSGs. **A**, **B** Differentially TFs are shown in a volcano plot (**A**) and a heatmap (**B**). Green, red, and black dots represent genes expressed at relatively lower, higher, or equal levels. **C** A regulatory network comprising differentially expressed TFs and DEOSGs. Triangles represent TFs, and red and green indicate risk and protective factors, respectively.** D** The respective protein-protein interaction (PPI) network analysis of PML, PYCR1 and MELK from TCGA datasets. The colored dot indicates a straight functional protein cluster to the three key genes. (https://string-db.org/, http://gepia.cancer-pku.cn/index.html). **E**, **F** The GO and KEGG pathway enrichment analysis of PML, PYCR1 and MELK-related partners. Significantly enriched pathways are indicated in *Y* axis. Gene Ratio in the *X* axis represents the enrichment levels. The larger value of Gene Ratio represents the higher level of enrichment. *KEGG*, Kyoto encyclopedia of genes and genomes; *GO*, gene ontology

To explore the potential regulatory mechanisms of transcription factors (TFs) and DEOSGs, we downloaded tumor-related TFs. Then, a total of 60 differentially expressed TFs were ultimately identified, including 41 upregulated TFs and 19 downregulated TFs (Fig. 3A, B). Moreover, we performed correlation analysis to predict the regulatory networks between these DEOSGs and differentially expressed TFs. Cytoscape software was used to visualize the TF-based regulatory networks. The construction of TF-based regulatory networks clearly revealed the regulatory relationships (Fig. 3C). Among the six DEOSGs, we focused on MELK, PYCR1, and PML, which have shown highly association with ccRCC patients’ outcomes. In order to decipher the underlying mechanisms by which the three key genes influenced tumorigenesis of ccRCC, we performed the PPI network analysis. As presented in Fig. 3D, the PYCR1-binding PPI networks, MELK-binding PPI networks and PML-binding PPI networks were created by the STRING online database. The GO enrichment analysis results revealed that the three key genes mainly correlated with immune response, immunoglobulin production and meiotic cell cycle. Moreover, the KEGG pathway analysis revealed 12 significantly enriched pathways, including cytokine-cytokine receptor interaction, cell cycle and neuroactive ligand-receptor interaction (Fig. 3E, F).

### Clinical prognostic analysis and validation of hub gene expression

**Fig. 4:**
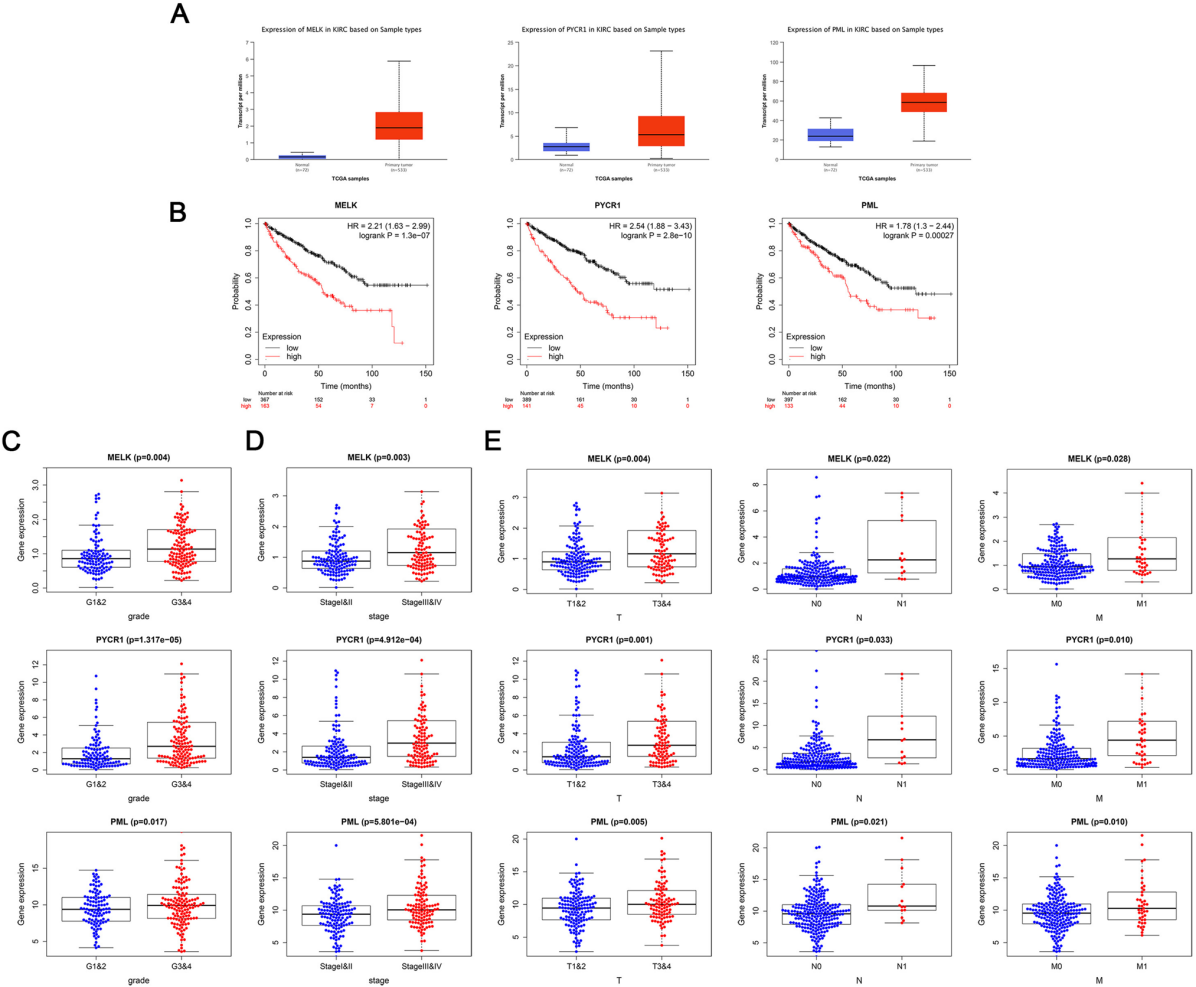
Clinical prognostic analysis and validation of hub genes. **A** Express levels of MELK, PYCR1, and PML in ccRCC tumor tissues or normal tissues in the TCGA database. **B** The overall survival curves of MELK, PYCR1, and PML according to the Kaplan–Meier Plotter database. **C**–**E** The association between the mRNA expression levels of MELK, PYCR1, PML and grade (**C**), stage (**D**), TNM stage (**E**)

First, we analyzed the expression levels of MELK, PYCR1, and PML according to the TCGA database, and we showed that the three hub genes were upregulated in ccRCC tumor tissues compared with matched normal tissues (Fig. 4A). Based on the Kaplan–Meier Plotter database, we determined the association of MELK, PYCR1, and PML with OS. The OS curves indicated that patients with higher levels of the three genes had worse outcomes (Fig. 4B). Moreover, we measured the association between the mRNA expression of the three key genes and different clinical characteristics, which showed that their mRNA expression was positively related to tumor pathological grade, stage and TNM stage (Fig. 4C-E).

### Immune infiltration analysis of MELK, PYCR1 and PML

**Fig. 5:**
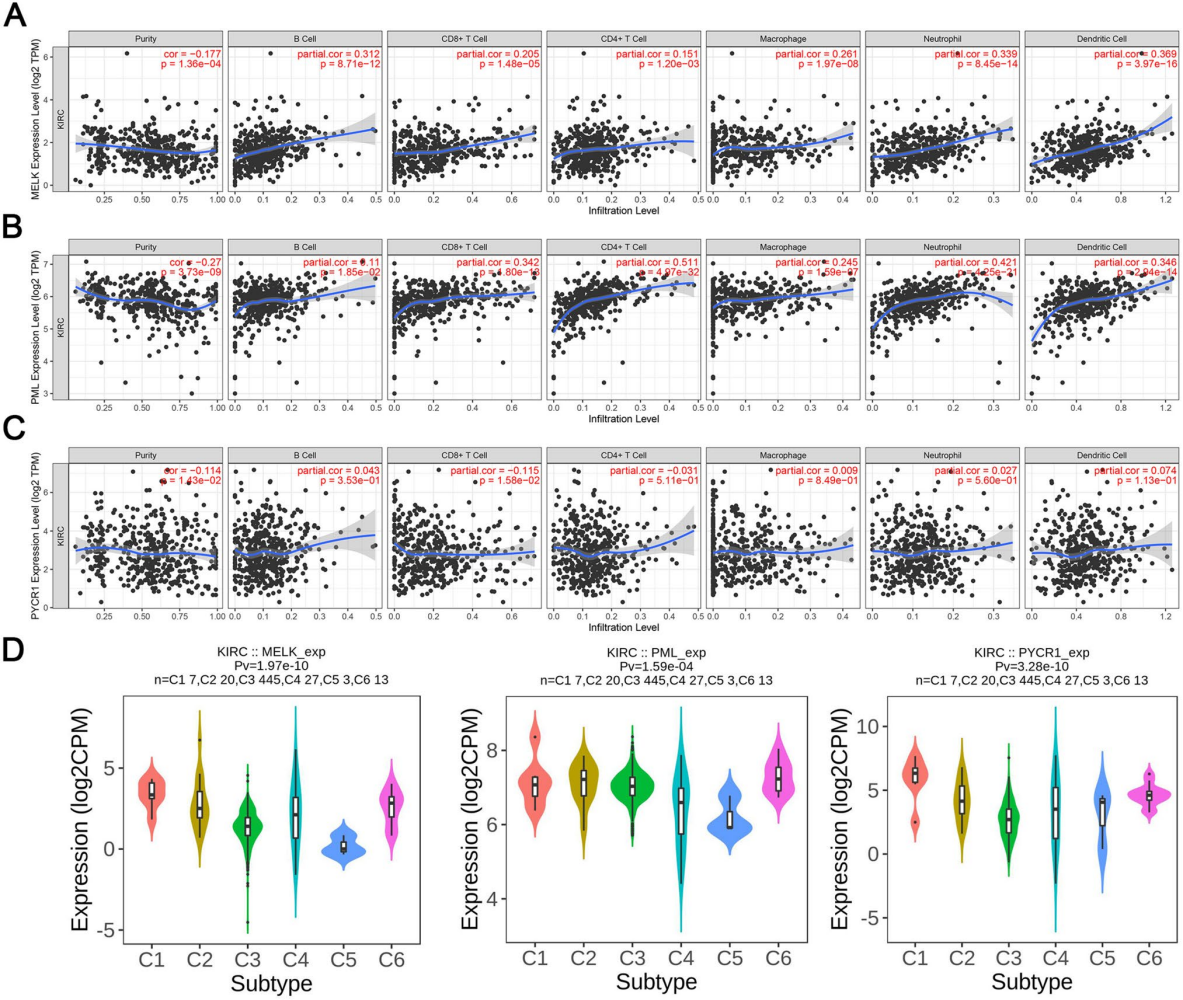
Immune infiltration analysis of MELK, PML and PYCR1. **A**–**C** The correlation between MELK (**A**), PML (**B**), PYCR1 (**C**) and immune cell infiltration using the TIMER database. **D** Distribution of MELK, PYCR1, and MELK mRNA expression in diverse immune subtypes of ccRCC according to the TISIDB database

To determine the capability of the prognostic model to indicate the status of the tumor immune microenvironment, we conducted a systematic exploration of the association between hub genes and immune cell infiltration based on the TIMER database. As shown in Fig. 5A–C, MELK demonstrated a significant correlation with dendritic cells (cor = 0.37), neutrophils (cor = 0.34) and B cells (cor = 0.31). In addition, increased expression of PYCR1 and PML was significantly correlated with CD4^+^ T cell, neutrophil, and dendritic cell infiltration (*P* < 0.05). Moreover, we analyzed the underlying relationships between the mRNA levels of the three key genes and the immune status of ccRCC (*P* < 0.05). The results from the TISIDB database showed that MELK, PYCR1, and PML expression was obviously altered between diverse immune status of ccRCC, which suggested that the three key genes were highly correlated with immune infiltration in ccRCC (Fig. 5D).

### Validation of elevated MELK and PYCR1 expression in ccRCC tissue by tissue microarray

**Fig. 6:**
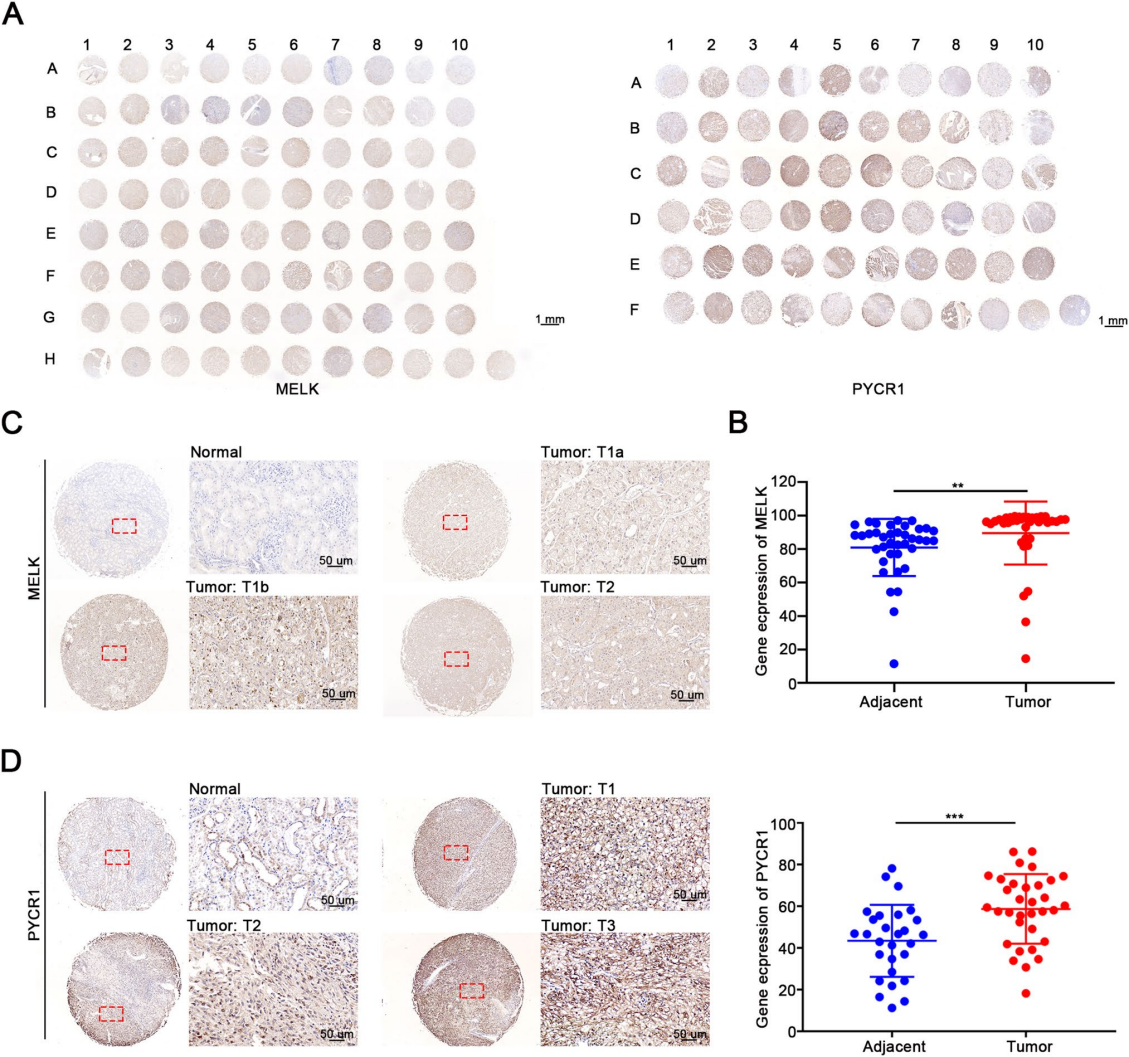
MELK and PYCR1 expression were elevated in ccRCC tissue by the tissue microarray. **A**, **B** The expression of MELK and PYCR1 in ccRCC tissues and adjacent normal tissues was detected by TMAs. **C**, **D** Representative views showed the IHC staining of normal tissues and tumor tissues including different clinical pathological stages. The enlarged images of MELK were selected from Figure A (normal corresponded to A10; Tumor T1a corresponded to D05; Tumor T1b corresponded to E09; Tumor T2 corresponded to F09). The enlarged images of PYCR1 were selected from Figure B (normal corresponded to A09; Tumor T1 corresponded to B04; Tumor T2 corresponded to F06; Tumor T3 corresponded to C06). Scale bar is 50 μm. **E**, **F** IHC score of MELK and PYCR1 expression showed the up-regulated levels in ccRCC tissues than benign renal tissues

To further determine whether MELK and PYCR1 expression was associated with clinical outcomes of ccRCC patients, we used a human ccRCC tissue microarray to perform an IHC assay. The specimens were collected from 38 ccRCC patients, including tumor tissues or adjacent tumor tissues, and stained for MELK **(**Fig. 6A**)**. Moreover, the PYCR1 microarray contained 28 samples of ccRCC tissues and matched tumor edge renal tissues **(**Fig. 6B**)**. As shown in Fig. 6E and 6F, the protein levels of MELK and PYCR1 were upregulated in tumor tissues compared with adjacent tissues. Additionally, the expression of MELK and PYCR1 was positively associated with the TNM stage of ccRCC patients **(**Fig. 6C, D**)**.

### Knockdown of MELK and PYCR1 inhibited ccRCC cell proliferation by elevating ROS levels and inducing cell cycle arrest in the G1 phase and apoptosis

**Fig. 7:**
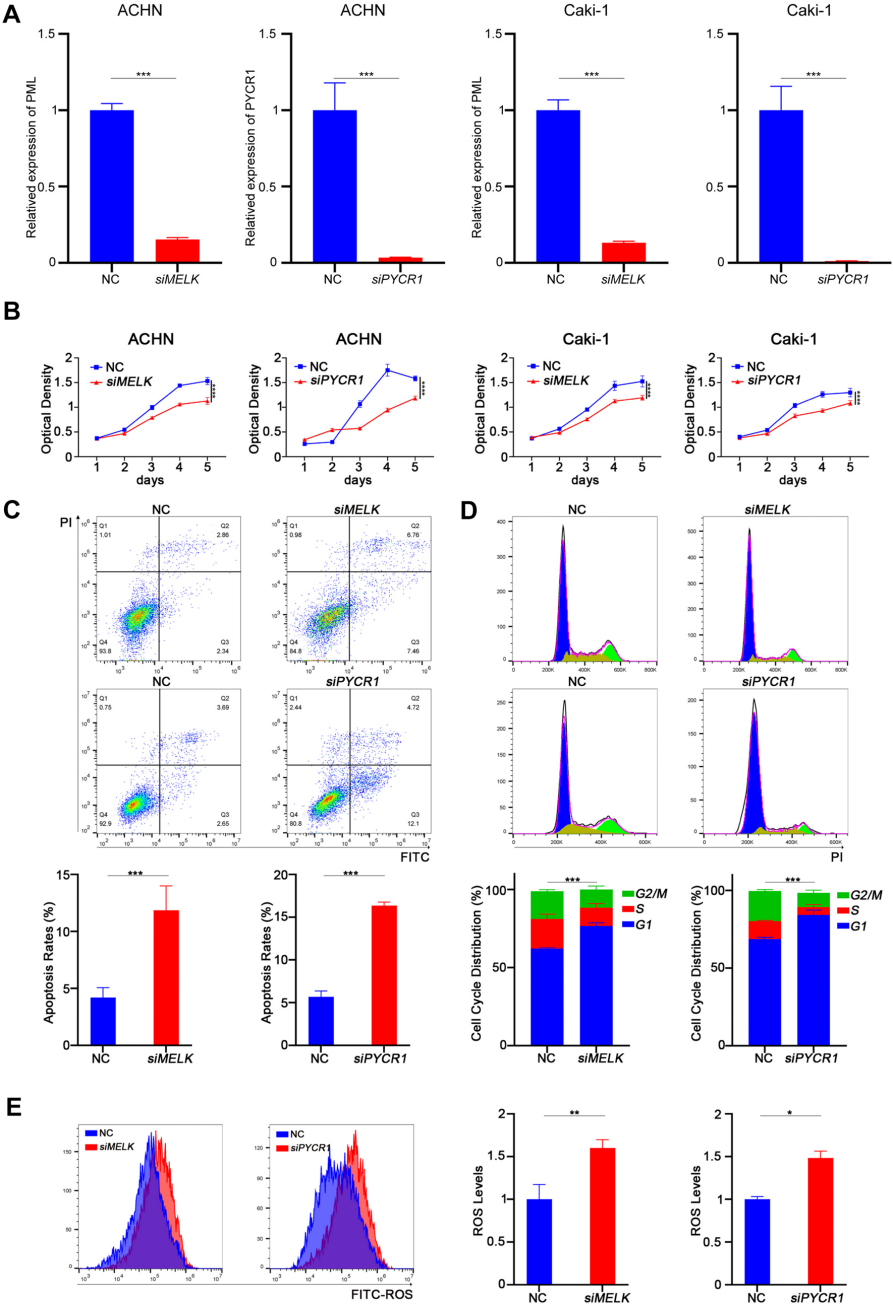
Knockdown of MELK and PYCR1 inhibited ccRCC cell proliferation by elevating ROS level, inducing G1 phase cell cycle arrest and apoptosis. **A** The mRNA levels of MELK or PYCR1 was weakened by si-MELK and si-PYCR1 transfection in Caki-1 and ACHN cell lines. **B** Knockdown of MELK or PYCR1 inhibited the proliferation of the ACHN and Caki-1 cell lines. **C**, **D** The si-MELK or si-PYCR1 induced cell apoptosis increasing, cell G1 phase cycle arrest, and added intracellular ROS levels **(E)** in Caki-1 cell line

To verify the biological functions of MELK and PYCR1 in ccRCC cells, we knocked down the expression of MELK or PYCR1 in the Caki-1 and ACHN cell lines, respectively (Fig. 7A). The MTT assay results demonstrated that the knockdown of MELK or PYCR1 inhibited cell proliferation in both ccRCC cell lines (Fig. 7B). Apoptosis assays and cycle assays also verified that *siMELK* or *siPYCR1* induced ccRCC cell apoptosis and cell cycle arrest in the G1 phase (Fig. 7C-D, Supplementary 7A-B). Moreover, the ROS levels were significantly increased after silencing the *MELK* or *PYCR1* genes according to flow cytometry (Fig. 7E, Supplementary 7C).

## Discussion

ccRCC is one of the leading causes of malignant urinary tumors, and its incidence is increasing year by year (Siegel et al. 2018). Despite recent improvements in surgical therapy for early ccRCC, patients with high-stage tumors still suffer from high metastasis rates and poor prognoses (Patard et al. 2011; Fisher et al. 2013). Therefore, the exploration of novel tumor biomarkers for the early detection, prognostic assessment, and appropriate therapy of ccRCC is extremely urgent.

Recently, ccRCC has been classified as a “metabolic disease” due to common alterations in cellular metabolic pathways that are associated with cancer initiation and progression (Linehan et al. 2010). The enhanced metabolic activity of cancer cells results in ROS overproduction, leading to changes in many processes that are necessary for tumor initiation, growth, and progression (Sullivan and Chandel 2014). In light of ROS overproduction, ccRCC tumors express high levels of antioxidants, such as reduced glutathione (GSH), to prevent the accumulation of multiple ROS (Bansal et al. 2019). Furthermore, increased oxidative stress is an important factor that leads to abnormal intracellular signal transduction (Kim et al. 2019). ROS can induce continuous activation of the pSTAT3, NF-κB and mitogen-activated protein kinase (MAPK) signaling pathways, regulate angiogenesis, accelerate tumor metastasis and promote the growth of ccRCC (Zhang et al. 2017). Due to the close association of ROS and their interactions with oxidative stress in ccRCC, it is necessary to research ROS-related biomarkers that can accurately predict clinical results and therapeutic targets.

In our research, 12 DEOSGs with prognostic value were screened, and an interaction network was constructed to identify the mechanism underlying the regulatory functions of these DEOSGs. Then, a multivariate Cox model was established to identify six prognosis-related hub genes, which were verified using statistical methods. The survival analysis revealed that the three key genes (MELK, PYCR1 and PML) could serve as independent prognostic factors. Furthermore, the results from the TIMER database elucidated that the mRNA expression of the three genes was strongly correlated with immune cell infiltration. These results demonstrated the prognostic value of a DEOSG-dependent risk model for patients with ccRCC. Moreover, the results indicated that these screened genes were significantly associated with ccRCC progression. However, previous studies have shown that PML plays crucial roles in the tumorigenesis of ccRCC (Lin et al. 2014). Therefore, we mainly focused on the search for PYCR1 and MELK and further confirmed that PYCR1 and MELK were critical for the comprehensive evaluation of the mechanism, which could be a novel direction for further research.

PYCR1 (pyrroline-5-carboxylate reductase 1), consisting of 10 exons, plays an important role in proline biosynthesis (Phang et al. 2015). Proline is a typical amino acid with secondary amines, and it is abundant in the cell microenvironment and is known as an indicator of various pathological stresses that occur during the process of tumorigenesis (Elia et al. 2017). However, as an active enzyme that catalyzes proline synthesis, PYCR might play significant roles in amino acid and energy metabolism, oxidative stress, and the malignant progression of some kinds of cancers (Chen et al. 2019). Under hypoxic conditions, the availability of proline is conducive to ATP generation or autophagy caused by ROS, which is essential for cancer cell growth and survival (Liu and Phang 2012). Due to these functions, proline may be associated with the abnormal expression of PYCR1 in cancer cells. Increasing evidence suggests that PYCR1 is highly expressed in various cancers, such as prostate cancer (Zeng et al. 2017), non-small cell lung cancer (NSCLC) (Cai et al. 2018) and hepatocellular cancer (Zhuang et al. 2019). For example, previous research demonstrated that silencing of PYCR1 inhibited proliferation, invasive migration capability, epithelial-mesenchymal transition, and metastatic abilities in hepatocellular carcinoma (HCC) (Guo et al. 2021). In addition, PYCR1 was also reported to be overexpressed in NSCLC and to promote the development of NSCLC by activating the p38 pathway (Wang et al. 2019). Consistent with previous studies on PYCR1 in other cancers, our findings predicted that PYCR1 was overexpressed in ccRCC tissues, which indicated a poor prognosis for ccRCC patients. In vitro cell function experiments further confirmed that knockdown of PYCR1 inhibited the proliferation of ccRCC cells. Additionally, flow cytometry showed that the silencing PYCR1 can increase ROS levels, as well as the proportion of cells that are arrested in the G1 phase. Furthermore, studies have shown that PYCR1 is closely associated with the TME, which has a distinct impact on tumorigenesis (Chen et al. 2021). ROS-related inflammatory cytokines, such as IL-13 and VEGF-A, can polarize macrophages toward an M2-like phenotype (Kuo et al. 2020). As the primary immunosuppressive cells in the TME, the M2-like phenotype of tumor-associated macrophages (TAMs) can give rise to a state of immunosuppression, which is conducive to the occurrence of tumors (Mantovani et al. 2002). In our study, we showed that PYCR1 was highly correlated with immune infiltration in ccRCC. Taken together, our data showed that PYCR1 may be a promising biomarker in ccRCC.

Maternal embryonic leucine zipper kinase (MELK) was first identified in *Xenopus* oocytes and embryos. It is a member of the AMP-activated protein kinase family of serine-threonine kinases (Thangaraj et al. 2020). In addition, the main posttranslational modification regulation of MELK relies on its 16 autophosphorylation sites (Seong et al. 2017). The inactivation of MELK is dependent on the direct phosphorylation of thioredoxin (Trx) at the Thr^76^ site (Manoharan et al. 2013). When MELK is activated by ASK1/TGF-β/p53 signals, Trx dissociates from MELK, and ZPR9 binds to the Thr^252^ site (Seong et al. 2002). As the focal point of several signal transduction pathways, MELK can regulate various bioprocesses, such as apoptosis, cell cycle arrest, and cell proliferation (Thangaraj et al. 2020). The earliest studies showed that MELK can directly phosphorylate and activate apoptosis signal-regulating kinase 1 (ASK1), which mediates the JNK and p38 signaling pathways and induces cell death (Jung et al. 2008; Seong et al. 2012). Moreover, MELK has been shown to regulate apoptosis and cell growth by affecting TGF-β-mediated signaling (Seong et al. 2010). Other studies have also shown that MELK can interact with p53 to regulate its function and affect normal cellular physiological functions (Seong and Ha 2012). Although MELK expression is tightly regulated in normal cells, it is significantly upregulated in a variety of tumor tissues (Thangaraj et al. 2020). Many studies have revealed that MELK performs tumor-promoting functions regarding the tissue origin of the cancer, and high expression of MELK is positively correlated with poor patient prognosis (Thangaraj et al. 2020; Lee et al. 2007; Martín-Martín et al. 2016). Recently, MELK has been defined as a crucial oncogene that plays indispensable roles in the Akt/mTOR signaling pathway to mediate the progression of colorectal cancer (CRC) (Tang et al. 2022). Moreover, MELK has been reported to be associated with the tumorigenesis of lung adenocarcinoma (LUAD) by increasing the migration and invasion of LUAD cells by regulating the EMT signal pathway. In recent years, an increasing number of potential therapeutic methods have focused on redox homeostasis in ccRCC, such as supplementation with antioxidants to target elevated ROS levels, which leads to the apoptosis of tumor cells (Mihailovic et al. 2021; Zacharias et al. 2021). Our current data also showed that downregulation of MELK could lead to an elevation in ROS generation. Whether MELK-related oxidative stress determines influences cellular homeostasis and the prognosis of ccRCC cells will require further investigation.

In the present study, we identified PYCR1 and MELK as prognosis-associated DEOSGs via a variety of bioinformatics analyses and verifications, and these genes were upregulated in human ccRCC clinical specimens in comparison to normal tissues. Moreover, in the TCGA database, the upregulation of PYCR1 and MELK was associated with late clinical features (grade, stage, TNM stage) and poor prognosis in ccRCC patients. In addition, based on the TIMER and TISIDB databases, we determined that PYCR1 and MELK were independently correlated with immune infiltration in ccRCC. This indicated that both genes may be important immune biomarkers and immunotherapy targets in ccRCC therapy. To verify the effects of PYCR1 and MELK on oxidative stress, we conducted an ROS assay to indicate that they play critical roles in the regulation of redox equilibrium. Inevitably, however, our study has some limitations. On the one hand, this study was based on open-source databases, and the clinical information was probably incomplete and limited. On the other hand, the specific mechanism underlying the change in expression of the two genes during oxidative stress in ccRCC still needs further study. We need to conduct further experiments to verify the specific biological mechanisms of PYCR1 and MELK as well as the tumor-immune axis in ccRCC. In conclusion, we generated a risk model comprising the ROS-related genes PYCR1 and MELK, which may contribute to regulating the development of ccRCC progression and serve as independent prognostic factors for patients with ccRCC.

## Conclusions

In summary, our present study analyzed and identified two crucial DEOSGs (PYCR1 and MELK) using integrated bioinformatics analysis. Then, we demonstrated the overexpression of PYCR1 and MELK in ccRCC and verified this finding by analyzing tumor specimens, and overexpression of these genes indicates a poor prognosis for ccRCC patients. These two novel biomarkers could provide us with refreshing insights into the diagnosis, progression and risk prediction of ccRCC patients. Furthermore, these findings could lead to the development of promising personalized and precision therapies combined with these two biomarkers for ccRCC.

## Supplementary Information

Below is the link to the electronic supplementary material.

Supplementary Figure 7. Knockdown of MELK and PYCR1 inhibited ccRCC cell proliferation by elevating ROS level, inducing G1 phase cell cycle arrest and apoptosis. (A) Knockdown of MELK or PYCR1 inhibited the proliferation of ACHN cell line. (B-C) The si-MELK or si-PYCR1 induced cell apoptosis increasing, cell G1 phase cycle arrest, and added intracellular ROS levels in ACHN cell line (TIF 17724 kb)

## Data Availability

The data that support the findings of this study are openly available in The Cancer Genome Atlas (TCGA) database at https://genomecancer.ucsc.edu/.
