# Supplementary figures and images for: Integrated analysis of the roles of oxidative stress related genes and prognostic value in clear cell renal cell carcinoma
Source: J Cancer Res Clin Oncol. 2023 Jun 20;149(13):11057–71. doi: 10.1007/s00432-023-04983-w (PMC10465389; doi:10.1007/s00432-023-04983-w)

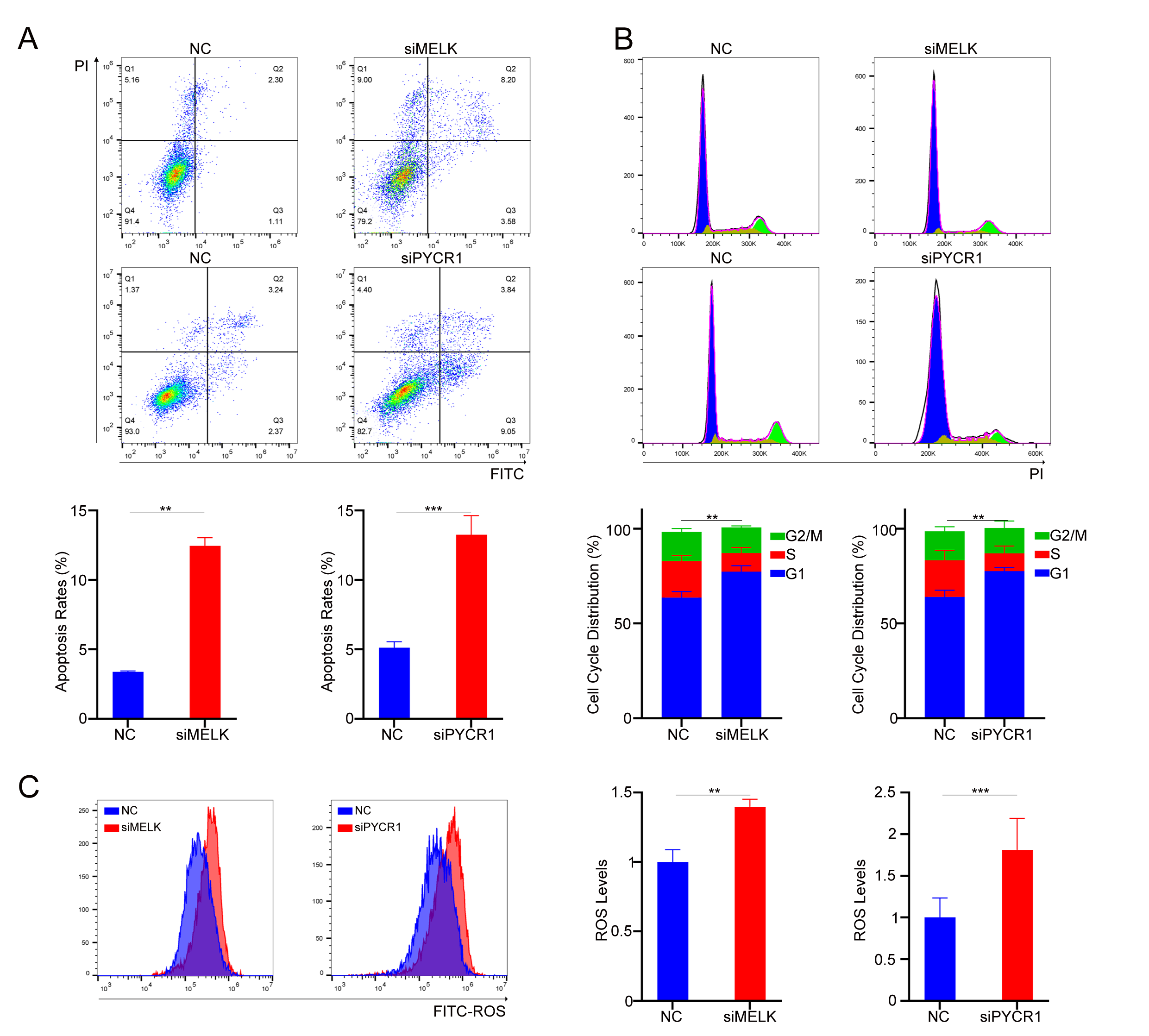

Supplement: Supplementary file 1 — Supplementary Figure 7. Knockdown of MELK and PYCR1 inhibited ccRCC cell proliferation by elevating ROS level, inducing G1 phase cell cycle arrest and apoptosis. (A) Knockdown of MELK or PYCR1 inhibited the proliferation of ACHN cell line. (B-C) The si-MELK or si-PYCR1 induced cell apoptosis increasing, cell G1 phase cycle arrest, and added intracellular ROS levels in ACHN cell line (TIF 17724 kb) [file 432_2023_4983_MOESM1_ESM.tif]
